# Rural mothers' beliefs and practices about diagnosis, treatment, and management of children health problems: A qualitative study in marginalized Southern Pakistan

**DOI:** 10.3389/fpubh.2022.1001668

**Published:** 2023-01-04

**Authors:** Farooq Ahmed, Najma Iqbal Malik, Sidra Zia, Abdul Samad Akbar, Xiaoyu Li, Muhammad Shahid, Kun Tang

**Affiliations:** ^1^Vanke School of Public Health, Tsinghua University, Beijing, China; ^2^Department of Anthropology, Quaid-i-Azam University, Islamabad, Pakistan; ^3^Department of Psychology, University of Sargodha, Sargodha, Punjab, Pakistan; ^4^Department of Anthropology, Islamia University of Bahawalpur, Bahawalpur, Punjab, Pakistan; ^5^School of Mathematics and Information Science, Xiangnan University, Chenzhou, Hunan, China; ^6^School of Insurance and Economics, University of International Business and Economics (UIBE), Beijing, China; ^7^World Health Organization Sub-office, Peshawar, Pakistan

**Keywords:** beliefs practices, health-seeking, magico-religious, rural mothers, Pakistan

## Abstract

**Introduction:**

Appropriate health-seeking beliefs and practices are indispensable for the survival and development of children. In this study, we explore childcare beliefs and practices of rural mothers and analyze the different ways childhood illness is diagnosed and managed in a marginalized rural community in Southern Pakistan.

**Methods:**

Using purposive sampling, in-depth interviews are conducted to obtain qualitative data from 20 illiterate and rural mothers in addition to 15 healthcare providers in the district Rajanpur of South Punjab.

**Results and discussion:**

The findings reveal that rural mothers' access to healthcare and therapeutic programs is impeded due to geographical isolation, structural inequalities, poverty, and illiteracy. Consequently, evil eyes, witchcraft, and spirits are recognized as potential threats to children's health and nutrition. Therefore, the treatment of childhood morbidity and malnutrition is mostly performed with folk, domestic, herbal, magico-religious remedies, and spiritual healing methods. The current study also highlights that many low-income and rural mothers tend to normalize childhood illness when they become unable to advocate for their children's health and nutrition. Besides improving low-income mothers' access to healthcare facilities, health education and risk communication at the field level through field health staff could be most effective for health promotion.

## 1. Introduction

The major causes of infant morbidity, mortality, and malnutrition in low-and-middle-income countries are complications related to preterm birth, intrapartum events such as birth asphyxia, and fatal infections such as diarrhea, and pneumonia (1). In Pakistan, especially in rural areas, the newborn and infant mortality rate is the world's third-highest (2). Healthier child-care practices benefit the brain development, growth, and overall health and nutrition of infants and children, however, they are lacking in much of rural Pakistan areas. A better understanding of how low-income, illiterate and rural mothers from underdeveloped and remote areas experience and manage illness might help improve these childcare practices. The public understands and responds to illness not only as an individual choice, but social disparities in health care contribute to a great extent to their everyday life (3). People's response to healthcare needs also depends on their access to the available resources, their belief in these healthcare sectors, and their understanding of the illness.

The reduction of child mortality rates has been a major focus of the Convention on the Rights of Children Initiative (4). The remote and underdeveloped areas often lack the services of the biomedical sector. Hence, their dependence on the popular and folk sectors alternatively compensates for their limited access to the specialized sector. Consequently, within the popular and folk sector, they incorporate their causative thinking to explain illness and treatment with reference to the magico-religious healthcare paradigm. The traditional magico-religious healing practices are healing rituals prescribing health-seeking behaviors concerning beliefs in supernatural and/or natural powers (5). The magico-religious medicines function as rituals, and folk medical practitioners such as religious healers play a significant role in traditional healing practices. Further, healing rituals are performed to seek God's forgiveness and favors that might result in cures and protection from the disease (6).

Evidence shows women from tribal and remote rural Pakistan hardly have access to different health and nutrition programs (7), which exposes multiple structural inadequacies. Also, females' mobility from one place to another is often restricted due to cultural norms. To exercise the right to healthcare in this scenario, what methods they should adopt, choices they could follow, and the rational decisions they should make depend upon paraphernalia of social, economic, and cultural opportunities. Because access to healthcare depends on socio-economic, psychological, and demographic background, people treat illness and malnutrition according to their social, economic, and cultural capital (8). Health-seeking behavior and socio-cultural environment are often interdependent. (9). The “inability to command commodities” and inaccessibility to better healthcare are strongly associated with disease and poverty (10).

In the Southern Punjab region of Pakistan, Rajanpur is one of the least developed districts with a high poverty rate. Nearly 60% of the total population is identified as poor and the human development level is the lowest of all districts within the Province of Punjab. The literacy rate is drastically low with only 11% in females and 29% in males. Underground water is brackish, especially in the western parts of the district. Therefore, residents use canals and rainwater as the primary source of drinking water, which often becomes the cause of diarrheal infections in infants and young children. Maternal-child malnutrition rates in the district are the highest in the whole province (11). Of the total population of the district (~2 million), 85% of households are rural. Only 17% of houses are built with concrete, 40% have electricity, 9% have piped water and ~1% of households use natural gas for cooking. The total number of Basic Health Units (BHU) is 33, Rural Health Centers (RHC) are 6, and 1 tertiary hospital with 40 beds. (12, 13). A long mountain range is on its West, and a great Indus River flows in the East the district face natural disasters of floods from two sides. The remote Western areas near to Suleiman Mountains are geographically secluded. The small roads connecting remote and small villages with the main highway are damaged. Adjacent to the main highways and having better access to biomedical healthcare facilities, the beliefs and practices of urban and peri-urban populations considerably differ from inhabitants of the tribal and remote rural areas.

Understanding the economic, social, cultural, and environmental context of these beliefs is useful for understanding local knowledge and incorporating interventions. This article attempts to explore the healthcare belief practices among women living in remote rural areas and deconstruct their sociocultural rationales behind these beliefs and practices. Why do rural mothers consider these cultural constructs important and avoid biomedical recommendations? To fill this gap, the current research navigates locals “explanatory models, interpolations, and rationalizations for their sociocultural knowledge, beliefs, and practices regarding the diagnosis and treatment of childhood illness.

## 2. Materials and methods

### 2.1. Data collection

This study collected qualitative data during ethnographic research exploring the sociocultural construction of child and mother malnutrition in the Rajanpur district of South Punjab between January and July 2017. All those stakeholders that were involved in the diagnosis, treatment, and management of children diseases were our potential respondents, for example, healthcare providers as well as pregnant and lactating mothers from remote rural areas having young children with some sickness. Using purposive sampling qualitative data were collected in three stages. First of all, five Key Informant Interviews (KII) with officials of the Health Department were contacted in the first stage. They provided very rich qualitative data on the day-to-day childcare knowledge, beliefs, and practices of local rural women. In the second stage, these key informants helped us further in introducing and communicating with Lady Health Workers (LHWs), Traditional Birth Attendants (TBAs), and spiritual healers, and a Focus Group Discussion (FGD) was arranged with 10 participants. In the next stage, these LHWs and TBAs further facilitated us in identifying local mothers (*n* = 20) from rural, and remote communities in the district.

We developed a semi-structured interview guide based on two things (1) searching keywords such as health beliefs and practices and health-seeking behaviors and (2) researchers” (FA and SZ) previous acquaintance with biomedical, and cultural childcare beliefs, and practices of local rural women. This guide was also pre-tested with a few mothers and further updated during fieldwork whenever new information was obtained. We conducted all the interviews in Seraiki, a local language. The study explored how childhood illness is diagnosed and treated at the local level. To participate in the study we informed almost 30 mothers about the study's nature to seek their will. However, 20 mothers finally agreed to provide their formal consent for this study. All in-depth interviews were conducted at respondents' places so that they could feel comfortable during probing. All interviews with the local rural mothers ranged between 1 and 2 h. Some limitations in study must be acknowledged. KII covers only a limited number of people and the results may not be representative for the women in the area. Also, some meanings could be lost in the process of translation into English. Moreover, we did not use any audio recordings for the interviews intentionally, keeping in mind the cultural norms and comfort of local respondents. As the perspectives were taken from multiple stakeholders, it is a strength of this research. The details of all respondents who provided qualitative data in this study are given in Table 1.

**Table 1:** Respondents information (*n* = 35).

**Description of interviews and discussion**	**No of respondents (*n*)**
In-depth-interviews with local rural mothers	20
Focus group discussion with lady health workers, traditional birth attendants, and spiritual healers	10
Key informant interviews with healthcare providers	5

### 2.2. Data analysis

**Figure 1:**
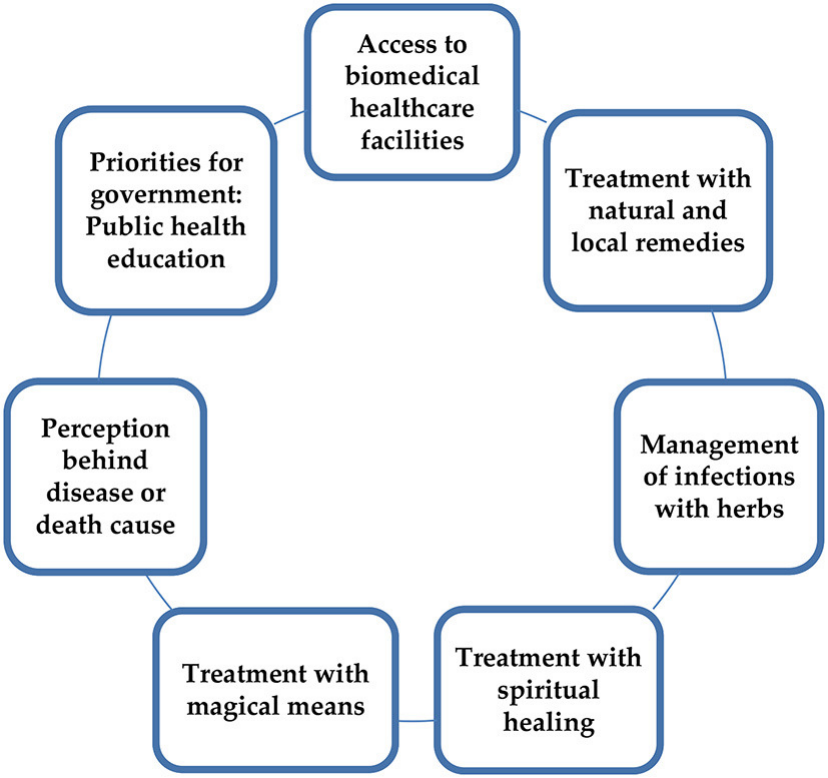
Disease etiologies and treatment practices among rural mothers in Southern Pakistan.

Field notes and interviews were immediately translated into the English language. After a rigorous evaluation of all translations, the qualitative data were investigated through a manual inductive analysis method. Noteworthy locals' narratives, accounts, and statements were noted down and analyzed by two co-authors who elicited different concepts, themes, and interpretations after comprehensive scrutiny of raw data. After cross-verifying all narratives and field observations, inconsistencies and analogous codes were separated with the joint consent of co-authors. We scanned the data and fragmented their meaning units with descriptive codes and conceptual categories. In the end, significant themes emerged from the qualitative data, which are as follows: (1) Mothers' access to biomedical healthcare facilities; (2) The perception behind disease or death cause; (3) Treatment through natural resistance and local remedies; (4) Management of infections with herbs; (5) Treatment with magic; (6) Seeking cure with spiritual healing methods; (7) Health education and priorities for the government (see Figure 1).

### 2.3. Ethical consideration

The Department of Anthropology and Advanced Studies and Research Board (ASRB) in its 307th meeting, of Quaid-i-Azam University (QAU) Islamabad, Pakistan approved this study and provided Ethical/IRB approval (Approval No. QAU-ASRB-2016-307; Date of approval 20-10-2016). We informed all respondents about the purpose and nature of the study before obtaining their free consent, and also strictly ensured their anonymity, privacy, and confidentiality after the data collection process.

## 3. Results

Social and demographic characteristics of participating mothers show that majority were below 35 years old, illiterate, had 3–8 children, and belonged to a lower socioeconomic status (Table 2).

**Table 2:** Sociodemographic characteristics of mothers (*n* = 20).

**Indicator**	**Frequency**	**Percentage**
**Mother age (in years)**		
15–20	2	10%
21–25	4	20%
26–30	5	25%
31–35	5	25%
36–40	4	20%
**Number of children**		
1–2	3	15%
3–5	10	50%
6–8	7	35%
**Maternal literacy level**		
Illiterate	16	80%
Primary-middle	3	15%
High or above	1	5%
**Occupation of mothers**		
Domestic or agricultural laborers	11	55%
Subsistence	7	35%
Other	2	10%
**Household income level (PKR) of mothers**		
< 5,000	2	10%
6,000–10,000	10	50%
11,000–15,000	5	25%
>16,000	3	15%

### 3.1. Theme one. Access to biomedical healthcare facilities and therapeutic program

The findings showed that low-income and rural mothers' access to public healthcare infrastructure often faced multiple structural inadequacies due to a lack of social capital.

“The majority of women in remote rural areas give birth at the hands of the Traditional Birth Attendants (dais). Quacks are also frequently consulted by the locals for the treatment of common health problems. Treatment at hospitals is often considered a last resort.” (Health Official 2, KII)

“The inhabitants of remote areas, especially females and pregnant women, have to suffer while being carried on some donkey cart, and most of them die en route to the closest health facility. In case of a severe ailment, many of the poor preferred treating their girls at the abode of a spiritual saint (Pir).” (Mother 18, IDI)

Criticizing expensive and dangerous biomedical practices, few mothers expressed their bad experiences with allopathic treatment methods. Some mothers stated the use of antibiotics was widespread among medical practitioners without tests. There was no difference in age while administering different types of medicines by doctors who prescribed various antibiotics simultaneously.

“For treatment, we often go to a doctor who runs the clinic near our home. He gives strong antibiotics. Last time my son got sick very seriously, so he was treated with multiple medications at the same time. Not sure what the ailment was, the doctor prescribed two or three antibiotics because he thought it might be either malaria or pneumonia. Though he is back to normal, my son is not as active as the rest of my children because of the excessive use of these antibiotics prescribed by the medical doctors. Because there is no check and balance from any higher authority doctors use antibiotics without fear.” (Mother 2, IDI)

“We are rural, illiterate, and poor people from remote villages. We can hardly become part of nutrition or therapeutic programs, especially when we are asked to stay at a stabilization center for the treatment of extremely sick children [complicated SAM]. First, long distances, traveling difficulties, then lack of contact with powerful people, and lastly poor mothers have to face stigma by health and nutrition staff in the hospitals. These are the reasons we cannot access programs and prefer alternative treatment at local places.” (Mother 7, IDI)

### 3.2. Theme two. The perception behind disease or death cause: Disease etiologies

As primary caregivers, rural mothers observe children's physical symptoms to diagnose the illness inferring possible causes according to their indigenous knowledge and beliefs. Traditional diagnosis and the supernatural causes behind sickness are the most common. There is a perception that a mother of a malnourished child could harm a healthy baby by casting an evil eye. Below are given some verbatims signifying common beliefs behind morbidity and malnutrition.

“For infants, shadow or reflection [of the mirror] is risky… if it comes onto the face or into the eyes of an infant during lactation…it causes diarrhea. Babies cry a lot… it becomes obligatory to neutralize it by an easy method… baby is passed through a round stand made of wood (Gharwanj), used to place mud pictures. Two women are required for this purpose, one above and the second underneath. One lady passes the infant to another lady through it and speaks loudly “should I drive out of reflection”… and the second lady catches the baby and says “yes do it.” So an infant disturbed with a shadow ultimately recovers.” (Mother 9, IDI)

“Our older women forbid drying babies' clothes on a suspended wire because the suspension on the wire could make their heads spin and they feel dizzy.” (Mother 20, IDI)

“Fear is shown when a child with a fair complexion wears a black shirt, the evil eye may hurt.” (LHW 1, FGD).

“Taking a small baby out of the room after sunset is disapproved. Malnutrition is perceived as a contagious disease, as the spirit of a malnourished child transcends from sick to a healthy child, So we often go to a spiritual healer for treatment through blowing.” (Mother 16, IDI).

“A pregnant woman must not encounter a lady who recently delivered a baby because, pollution of delivery could affect the pregnancy and for prevention, a mother ought to hide herself for one lunar month.” (LHW 3, FGD)

“Once I was pregnant, I heard that one of my female relatives has delivered a baby boy. The news made me glad and I rushed for congratulating her and seeing the baby. As I entered her room, I was stopped “why have you entered”… you are pregnant… delivery's pollution will hurt your pregnancy…now hide and avoid encountering delivering mother for at least one month.” (Mother 15, IDI)

“My husband's brother's wife (dewarani) did black magic (Kala Jadu) on my son. The main reason she used to cast it [black magic] on my deceased son was that she was jealous of my being as a mother of sons because she had daughters only. Because of this, “jadu-tona (black-magic)” my young son died.” (Mother 14, IDI)

“What a mother eats, it affects the child” is another local concept that commonly prevails.

“If a child is suffering from diarrhea, the mothers should become preventive in this regard. Mother is the chief source [of infection] and should avoid eating harmful foods.” (TBA 2, FGD)

### 3.3. Theme three. Treatment through natural resistance and local remedies

Because of limited access to both public and private medical healthcare, many mothers informed that they often preferred domestically treating their children with organic solutions.

“Our babies become resistant and tough to fight disease when parents become indifferent to care and cure. Vaccination is rare among us… as it makes children dependent.” (Mother 1, IDI)

“Ignoring the biomedical treatment of minor illnesses is normal among low-income households. If they developed some minor injury, they try to treat it on the spot, mostly with dirty things such as mortar or muddy water, with the belief that there is a healing essence in the dirt.” (Health Official 3, KII)

“Diseases are often treated domestically. For example, burning wheat or wood and its hot juicy liquid oozing out of wood's reverse is prescribed for the treatment of ringworm.” (Mother 8, IDI)

“If someone has Asthma, a burning scar resembling a thumb is given behind the neck or on the forehead.” (Mother 19, IDI)

“Every third day of chickenpox, the child is given a milk sprinkle, and on day seven, the baby took a bath. Belief in “milk as sacred” brings fortune to the sufferer.” (Mother 3, IDI)

“Early morning spit on injuries and sores is often recommended because it acts as an antiseptic.” (Mother 11, IDI)

### 3.4. Theme four. Management of infections with herbal methods

Results revealed that herbal methods were also very much common among rural mothers. They treated diseases and infections with different plants and herbs because these were inexpensive and available locally as compared to expensive biomedical medicines.

“Infants are introduced to cow's milk in the early six months, which often causes severe diarrhoeal infection. We treated it with local traditional tips. We give Pakka-Pani, which is made with water boiled with fennel, cinnamon, cardamom, and mint.” (Mother 12, IDI)

“We roast tankan borax (suhaga) in a frying pan, which swells after a while and rises. We grind it and mix it with bamboo exudate (tabasheer) and water and give a spoon full to the sick baby twice or thrice a day. The ill baby fully recovers.” (Mother 10, IDI)

“Tabasheer is also used to treat the baby's infected mouth along with a jet of fresh milk from a goat. We use to grind bishop's weed or carom (ajwain) and fennel (sounf) to treat the baby's belly pain.” (Mother 6, IDI)

“We treat diarrhea with castor oil… it results in watery stool for the first time and cleaning all dirty materials from the child's body. We believe that this is safe and proven. Low excreta is a sign that the child has become normalized.” (Mother13, IDI)

“We cure children at home. For stomach problems, we mix paneer and crystallized salt (noushadar) in the milk and give light heat. Milk spill, separate yogurt from golden water, and then put some almond oil in it. Another cure is to mix rose flower and fennel, give heat, gas will pass within seconds, and it cures pain in the belly.” (Mother 4, IDI)

“If the child is suffering from pneumonia I use a homemade syrup (Sehat) made with pure and natural things like jaggery, honey, Indian rennet (paneer), fennel, bitter wood (musag), and sweet wood (malathi).” (TBA 1, FGD)

### 3.5. Theme five. Treatment of diseases and illnesses in magical ways

Magic was another most popular method rural and illiterate mothers used for the treatment of severe illnesses and malnutrition in their infants and young children. One of the traditional treatments for low weight and wasting is the magical law of contradiction as it was perceived to be caused by black magic or the evil eye.

“Mother's physical closeness to a child can protect her/him from fear. We advise keeping milkweed plants near the malnourished child and spinning a tori (vegetable like brinjal) all around the sick and weak baby and throwing it on the roof.” (TBA, 1).

“Gold paper and tree leaves are put in a small bag; every Sunday this bag is spun, seven times around a malnourished child, and thrown into a well. It is repeated seven times a week and when it starts opening in water, the child is perceived to be recovering from illness.” (TBA 2, FGD)

“Eggplant or Brinjal for this ailment is also used. If it shrinks and becomes dry, the child will get nourished. Hugging the bitter milkweed (Uqq) plant is also prescribed as a medicine so that when the child grows, the plant bows down.” (Mother 17, IDI)

“My son was suffering from [kwashiorkor], a swelling belly, weak legs, and an enlarged head. He was treated but the baby could not be cured. Then some other person suggested that an amulet be written on an egg's shell and buried under the baby's bed. This method worked, and the sick baby grew up and recovered as long as the egg shrank. In one month, the baby started to walk and run and became perfect and healthy.” (Mother 18, IDI)

“An infant or young child should wear a metal ring around one hand's finger and a black color thread around the ankle for forty days. If the mother has to leave her baby for some time, she should keep her shirt with him; otherwise, the baby will be scared. Mother can also sprinkle her breastmilk on the bed.” (TBA 2, FGD)

### 3.6. Theme six. Treatment of children with spiritual healing methods

As diseases are perceived as spiritual therefore many locals prefer treatment with religious symbols. Also, those names and personalities who are considered most authoritative, sacred, and supernatural are often used for treatment and healing purposes.

“A vast majority of indigenous people avail themselves of other options such as visiting spiritual healers. Few ladies possessing spiritual powers are famous for treating different illnesses through recitation (salwaat).” (LHW 2, FGD)

“To cure a weeping and disturbed child, dum is often recited. Dum is a recantation of either religious or sacred text. Someone's bad eye is believed to make a baby sick and salwaat is the perfect cure for the evil eye.” (LHW4, FGD)

“During the recitation of salwaat, blowing air through the mouth is done continually coupled with spitting on the lesions. When these sufferers heal through prayer, they come again for paying their gratitude and thanksgiving, often with some gifts including a goat, sheep, or hen.” (Mother 5, IDI)

Verses of Throne are recited for the multi-purpose security of children. It is believed that the powers of the throne verses of the Quran and prophetic journey to the skies (miracle) annihilate every suffering. It is believed that when throne verses are recited seven times, they become a shield all around the child to protect them from danger, the black eye, and other crises.

“Verses of Throne are Eastern epistemology, Verses of Throne are deen and Quran; Seven skies, one court; Prophet mount up and ride please, armor and shield will kill seventy sufferings.” (Spiritual healer 1, FGD)

Also, the reciter gives intercession (*wasta*) of holy personalities to diseases. A *Salwaat* for breaking black magic is recited 21 times a day and for 21 days in total. It is perceived that black magic would return to the original place from where it originated.

“Rock, rock, rock, fence of rock; knot and hold it outside, filled with rocks everywhere; all kinds of magic rebound and bounce back toward; everyone who does it will die).” (Spiritual healer 2, FGD)

### 3.7. Theme seven. Priorities for government: Prevention education and promotion

Respondents of the study urged that government should take necessary measures for the development of rural areas, especially the health education movement for illiterate mothers.

“Priorities of decision-makers must be changed, there were missed priorities like clean drinking water supply, sanitation, and the government should have hit the right priorities, and avoid wrong such as megaprojects.” (Health Official 3, KII)

“Poor lacked the necessary facilities and the right living conditions. There was a lack of knowledge regarding treating infections. The health-seeking behavior of the illiterate and impoverished was not active, as they could not respond to any medical condition on time, which caused many casualties.” (Health Official 2, KII)

“Mothers are uneducated; we have to teach them a basic level of guidance, as to what is to use, when and how much dose is necessary. Many mothers pick up at once, but several others fail to put it in mind.” (Health Official 4, KII).

When asked by a senior medical officer, what mothers should do for effective management of different medical conditions? He informed:

“Mothers must be given primary first aid education to cope with common illness onset so that they can do some sort of self-control mechanism. Mothers should know that acetaminophen and ibuprofen are for pain; the antiseptic solution is for injuries and cuts. In case of diarrhea, the mother should know water, salt, and sugar levels of the child's body must be sustained, for this, she should use Oral Rehydration Salts (ORS), or she can make herself at home remedy if vomiting and motion are more than three times in a day. Mix one teaspoon of salt and two spoons of sugar in boiled water equal to 1 liter and use for 24 hours. Similarly, mothers must be knowledgeable about the symptoms of pneumonia if the ribs are moving faster than usual in 1 minute, the child should be taken to a doctor for treatment. The child's fever should be kept low by taking off warm clothes and making the feet and armpits of the child wet with fresh water.” (Health Official 5, KII)

“The poor are often stigmatized that they deliberately ignore biomedical treatment. The social inequalities compel mothers to adopt alternative therapies (other than biomedical) because they are inexpensive, easily accessible, and psychologically naïve enough. The role of development and equity or access of the poor to it is crucial. For the success of health and nutrition intervention, the required level of education is crucial, especially for mothers who deal with children, and understanding of the causal link. If the public is unaware of the mechanism and causal pathways, there are fewer chances to secure the expected results from a program that is conceived to make significant impacts.” (Health Official 1, KII)

## 4. Discussion

This study explored rural mothers' diagnostic beliefs and treatment practices for morbidity and malnutrition. Mothers from remote rural areas revealed that their access to biomedical healthcare facilities as well as therapeutic programs for the treatment of severe acute malnutrition was restricted due to social, economic, and structural inequalities. Evidence demonstrates that social and economic constraints among rural women in developing countries obstruct their access to healthcare, often due to geographical isolation, healthcare provider shortages, lack of appropriate funding, and lack of health education (14). Also, a study from Southern Punjab Pakistan indicates that a lack of cultural and social capital plays a negative role and becomes the cause of their social exclusion (15).

Findings show that alternative or traditional methods were common. Children at first were treated domestically. Grandmothers and mothers usually treated diarrhea at home according to their experience and knowledge. Almost similar traditional beliefs and practices exist in rural African communities (16). Also, low-income mothers of sick and malnourished children visit either a low-quality public health facility or the abode of spiritual healers for health-seeking. In addition to herbal treatment that is based on the family's traditional medicine learned from ancestors, other healing and protective measures included faith healing and secular magical practices. Magico-religious healing beliefs and practices are a significant aspect of health-seeking behavior among rural mothers. Literature has shown that culture determines the belief system of health and illness (17). Therefore, the health-seeking behavior of people is heavily influenced by these cultural belief systems. A study from South Africa similarly showed that reliance on traditional herbs, the notion of witchcraft, and faith healing determined treatment methods in children (18).

To understand health-seeking behavior, one's perception of illness needs to be explored (19). Our research indicated that the locals' prototypes of explaining and analyzing were analogous to binary opposed linguistic concepts and identical to magical realities like the “law of contradiction” or “law of similarity” (20, 21). They understood that diseases were transferred through a spirit from one body to another, and in this etiology of diseases the medium was a spirit, therefore, mystical treatments through magico-religious, and spiritual healing methods might best work (22). Thus, beliefs in the etiology of disease might have impacted their decisions about care and treatment-seeking (18).

Some mothers believed breastmilk from the impure body of the mother makes the child sick (23). A combined amalgamation of factors such as locals' environment, economy, culture, and religion provided a unique socio-cultural environment in which dichotomous explanatory frame of “sweet-sour,” “hot-cold,” and “purity-impurity” overshadowed empirical analytics, which seeped deeper into their beliefs and behaviors to eventually influence their carefulness, medication, and health-seeking practices (24). The causes of illness in other South-Asian countries, such as India and Bangladesh, are also perceived by both spiritual and pathological functions. Therefore, a combination of modern and traditional modes of treatment is employed, where elders, religious and traditional healers influence gender and structural forces construct disease perception and the choice of treatment (25). It is consistent with the evidence that chances of childhood morbidity and mortality increased due to their living in an extended family structure in which relatives, grandmothers, and family elders influence diagnosis and treatment decision-making (26).

The results found that the poor often normalized childhood diseases. Empirical inquiry about the causes of disease and death is postponed owing to the belief that worries can come and go with the will of God. A previous study in South Africa indicated that managing illness with prayers was preferred over western medicines as in their opinion God is more powerful than everything else in the universe (18). This finding is consonant with a previous study from Pakistan, which found that symptoms of illness were generally taken normally; therefore, there is no need to be sensitive about seeking care for treatment, and health-seeking behavior was strongly dependent upon people's belief systems relevant to their socio-cultural milieu (27, 28). There were other reasons too that compelled mothers to treat them at home first. The absence of metalled roads, lack of interest of household males in treatment, and low social, cultural, and economic capital were the most prominent reasons.

The local belief is that because childbirth involves the concept of pollution a pregnant lady should not encounter a mother who recently delivered a baby. As women might be a target of attack by malicious spirits during such periods, ladies ought to maintain purity refraining from pregnancy and birth-related dirt. The notion of purity and pollution has strongly influenced the perceptions in South Asia (29). In Northwestern Pakistan, people fear that a mother with impurity could catch a shadow (*saya*) and transfer it to the baby through her breastmilk (30, 31). Our results indicated that a shadow was treated by a spiritual healer. Similarly, studies from South Africa showed that the spirit of a child was affected by a dark entity so a spiritual healer can only do this. Traditional healers pointed out that the cause of the illness was spiritual (ancestors were angry to revenge from the mother) (18). Traditional healers understand locals' psychology and intrahousehold politics very well and predict accordingly as one mother illustrated how at the time of final burial the cause of his son's death was revealed by a traditional healer.

Some of the regional literature (32) informed about the childcare belief practices among rural Punjab mothers (33). These studies provide a magico-religious healthcare paradigm integrating magical thinking and the religious manifestation of childcare belief practices. Our findings indicated that mothers believed that certain vegetables would become dry, and the child might become fat. This is consistent with a study conducted in South Punjab in a Punjabi community (34) that shows a resemblance between an infant's healthcare belief practice in Seraiki and Punjabi communities living in rural Punjab.

Local perception about the contagion effects of diseases lies in their belief in evil spirits, evil eyes, and the tendency of the woman experiencing impurity (mensuration or birth pollution), or an evil sickness to bring harm to other healthy women or children. Therefore, a belief in magico-religious healing supersedes the treatment at a clinic that they see as ineffective unless the evil effects are not waived off. Evidence from Africa also showed that locals perceived the child's illness did not need medical attention because it was caused by spiritual reasons so consulting a traditional healer is better. South Africans considered that Supernatural causes such as curses and witchcraft were responsible for illness (18) and misfortune (35), which necessitated shopping for traditional healers Belief in the power of God and faith play a key role in deciding what type of medical care is required (36).

A qualitative study from the Sindh province of Pakistan also brings forth similar findings as to how rural communities seek religious and spiritual ways of health. According to this study, the spiritual healer treats illness through chanting and blowing and by giving amulets to wear in rural areas (37). In rural Punjab local beliefs, even spiritual and home remedies are tried in critical cases (38). Herbs are considered harmless and beneficial for immunity boost (39). However, such methods often delay health-seeking for children increasing their chances of morbidity even for easy-to-manage symptoms and conditions (40). A study showed that the use of traditional herbal concoctions caused child mortality (41). Literature showed locals had a lack of trust in western medicine as several side effects were attached to conventional medicine. Some recent studies in Southern Punjab concluded that low public health education and nutrition awareness in adjacent marginalized communities increased episodes of infections and malnutrition prevalence in under-five children (42, 43). Locally situated analyzes of coping strategies disclose that there are manifold coatings of sociocultural reality, which shape responses to illness and survival strategies. The behavior and psychology of rural mothers are closely affiliated with social structure. Rural female's health-related beliefs and practices can be improved in multiple ways. Education might be one solution for mothers in the rural setting. However, educating service providers might be also important since mothers felt stigmatized in a healthcare setting for not having enough money, literacy, social capital, etc. Service providers could use these results to improve their action plan and healthcare system strengthening at a rural setting.

## 5. Conclusions

This study finds that low-income and rural mothers in Southern Punjab face several social and structural vulnerabilities that restrict their social inclusion and access to healthcare and nutrition programs in the deprived areas of Southern Punjab. Low-income and illiterate mothers' perceptions of disease etiologies and responses to childhood diseases are constructed by socio-cultural conditions. The rural mothers' analysis of morbidity and malnutrition looks like dichotomous and binary-opposite linguistic realities such as sacred-profane, hot-cold, and purity-impurity. Therefore, powers of magic and religion bridge this gap through supernatural means of treating illnesses leaving the physical/medical illness untreated and putting the child's life at stake. We conclude that maternal literacy and public health nutrition education might be the most significant for improving children's health in remote, and rural areas of Southern Pakistan.

## Data availability statement

The original contributions presented in the study are included in the article/Supplementary material, further inquiries can be directed to the corresponding author/s.

## Ethics statement

The studies involving human participants were reviewed and approved by Quaid-i-Azam University Islamabad. The patients/participants provided their written informed consent to participate in this study.

## Author contributions

FA designed the study, conceived the manuscript, and drafted the manuscript. FA and SZ collected and analyzed data. NM, SZ, AA, XL, MS, and KT were involved in revising the manuscript. All authors were involved in writing the manuscript and approved its final version.

## References

[1] Victora CG Christian P Vidaletti LP Gatica-Domínguez G Menon P Black RE. Revisiting maternal and child under nutrition in low-income and middle-income countries: variable progress towards an unfinished agenda. Lancet. (2021) 397:1388–99. 10.1016/S0140-6736(21)00394-9 33691094 PMC7613170

[2] Ahmed F Leghari IU Shahid M Ahmad M. Newborn care practices: locals' analytical models, and potential medical risks in South-Punjab, Pakistan. Rawal Med J. (2021) 46:442–5.

[3] Parker RG Scheper-Hughes N. Death without weeping: the violence of everyday life in Brazil. Hisp Am Hist Rev. (1995) 75:714. 10.2307/2518093

[4] Tait CA Parnia A Zewge-Abubaker N Wong WH Smith-Cannoy H Siddiqi A. Did the un convention on the rights of the child reduce child mortality around the world? An interrupted time series analysis. BMC Public Health. (2020) 20:707. 10.1186/s12889-020-08720-7 32423476 PMC7236469

[5] Sexton DJ Ralph Corey G. Rocky mountain “spotless” and “almost spotless” fever: a wolf in sheep's clothing. Clin Infect Dis. (1992) 15:439–48. 10.1093/clind/15.3.439 1520791

[6] Andrews M Boyle JS Collins J. Transcultural Concepts in Nursing Care. J Transcult Nurs. (2002) 13:178–80. 10.1177/10459602013003002 12113145

[7] Bhutta ZA Hafeez A. What can Pakistan do to address maternal and child health over the next decade? Health Res Policy Syst. (2015) 13:13–6. 10.1186/s12961-015-0036-5 26792061 PMC4895255

[8] McGrath P Rawson N. Key factors impacting on diagnosis and treatment for vulvar cancer for Indigenous women: findings from Australia. Support Care Cancer. (2013) 21:2769–75. 10.1007/s00520-013-1859-7 23720063

[9] Obeyesekere G. 4. Depression, buddhism, and the work of culture in Sri Lanka. In: Kleinman A Good B, editors. Culture and Depression. Berkeley, CA: University of California Press (2020). p. 134–52. 10.1525/9780520340923-007

[10] Sen A. Poverty and Famines: An Essay on Entitlement and Deprivation. New Delhi: Oxford University Press (2013).

[11] Punjab Bureau of Statistics. Multiple Indicator Cluster Survey Key Findings Report. Lahore (2014). Available online at: http://bos.gop.pk/finalreport (accessed March 20, 2018).

[12] Ahmed F Shahid M Cao Y Qureshi MG Zia S Fatima S. A qualitative exploration in causes of water insecurity experiences, and gender and nutritional consequences in South-Punjab, Pakistan. Int J Environ Res Public Health. (2021) 18:12534. 10.3390/ijerph182312534 34886260 PMC8657084

[13] National Institute of Population Studies. Pakistan Demographic and Health Survey 2017–18: Islamabad, Pakistan, and Rockville; NIPS; ICF: Islamabad, Pakistan (2019). Available online at: https://dhsprogram.com/data/dataset/Pakistan_Standard-DHS_2017.cfm?flag=1 (accessed May 11, 2020).

[14] Behera BK Prasad R Behera S. Healthcare Strategies and Planning for Social Inclusion and Development: Volume 2: Social, Economic, and Health Disparities of Rural Women. Cambridge, MA: Elsevier (2022). 10.1016/B978-0-323-90447-6.00005-9

[15] Ahmed F Malik NI Malik N Qureshi MG Shahzad M Shahid M. Key challenges to optimal therapeutic coverage and maternal utilization of CMAM program in rural Southern Pakistan: a qualitative exploratory study. Nutrients. (2022) 14:2612. 10.3390/nu14132612 35807795 PMC9268698

[16] Mothibe ME Sibanda M. African traditional medicine: South African perspective. Tradit Complement Med. (2019) p. 1–27. 10.5772/intechopen.83790

[17] Spector RE. Cultural diversity in health and illness. J Transcult Nurs. (2002) 13:178–80. 12113150 10.1177/10459602013003007

[18] Mabetha K De Wet-Billings NC Odimegwu CO. Healthcare beliefs and practices of kin caregivers in South Africa: implications for child survival. BMC Health Serv Res. (2021) 21:1–12. 10.1186/s12913-021-06357-9 34022877 PMC8140432

[19] Rivers WHR. Medicine, Magic, and Religion: The Fitzpatrick Lectures Delivered Before the Royal College of Physicians of London in 1915 and1916. NewYork, NY: Routledge; Taylor and Francis (2001).p. 136.

[20] Rozin P Nemeroff C. Sympathetic magical thinking: the contagion and similarity “heuristics.” In Heuristics and Biases: The Psychology of Intuitive Judgement. Cambridge, MA: Cambridge University Press (2001). p. 201–16.

[21] Ahmed F Leghari I Alam MB Shahid M. 2019, Undefined Sociocultural Construction of the Ritual and Practice of Ghutti (Pre-lacteal): An Ethnographic Study in Rajanpur, Punjab Pakistan. Available online at: annalskemu.org (accessed December 8, 2020).

[22] Hurtado E. Breast feeding in the etiology of diarrhea. Arch Latinoam Nutr. (1989) 39:278–91. 2490882

[23] Bandyopadhyay M. Impact of ritual pollution on lactation and breastfeeding practices in rural West Bengal, India. Int Breastfeed J. (2009). 10.1186/1746-4358-4-2 19323839 PMC2667394

[24] Anderson EN. Why is humoral medicine so popular? Soc Sci Med. (1987) 2:331–7. 10.1016/0277-9536(87)90271-1 3317873

[25] Begum F. Women'*s Reproductive Illnesses and Health Seeking in a Bangladeshi Village*. University of British Columbia, Vancouver, BC, Canada. (2012).

[26] Gao M Brännström L Almquist YB. Exposure to out-of-home care in childhood and adult all-cause mortality: a cohort study. Int J Epidemiol. (2017) 46:1010–7. 10.1093/ije/dyw295 28031308 PMC5837321

[27] Nayab D. Health-seeking behaviour of women reporting symptoms of reproductive tract infections. Pak Dev Rev. (2005) 44:1–35. 10.30541/v44i1pp.1-35

[28] Anwar M Green J Norris P. Health-seeking behaviour in Pakistan: a narrative review of the existing literature. Public Health. (2012) 126:507–17. 10.1016/j.puhe.2012.02.006 22579325

[29] Rozario S Samuel GB. Tibetan and Indian ideas of birth pollution: similarities and contrasts. In Daughters of Hariti. London: Routledge (2020). p. 193–219.

[30] Mull DS. Traditional perceptions of marasmus in Pakistan. Soc Sci Med. (1991) 32:175–91. 10.1016/0277-9536(91)90058-K 1901666

[31] Mull DS. Mother's milk and pseudoscientific breast milk testing in Pakistan. Soc Sci Med. (1992) 34:1277–90. 10.1016/0277-9536(92)90320-P 1641686

[32] Qamar AH. Belief in the evil eye and early childcare in rural Punjab, Pakistan. Asian Ethnol. (2016) 75:397–418.

[33] Hameed QA. The postpartum tradition of Sawa Mahina in Rural Punjab, Pakistan. J Ethnol Folk. (2017) 11:127–50.

[34] Qamar AH. Tona, the folk healing practices in rural Punjab, Pakistan. J Ethnol Folk. (2015) 9:59–74. Available online at: https://www.jef.ee/index.php/journal/article/view/194 (accessed December 16, 2022).

[35] Paulk ME. Understanding the role of religion in medical decision making. J Oncol Pract. (2017) 13:219–20. 10.1200/JOP.2016.020693 28287853

[36] Peprah P Gyasi RM. Religion and health in urban Ghana. BMC Public Health. (2018) 18:1394. 10.1186/s12889-018-6277-9 30572873 PMC6300874

[37] Premji S Khowaja S Meherali S Forgeron R. Sociocultural influences on newborn health in the first 6 weeks of life: qualitative study in a fishing village in Karachi, Pakistan. BMC Preg Child Birth. (2014) 14:1–12. 10.1186/1471-2393-14-232 25030836 PMC4223389

[38] Qureshi K Qureshi A Khawaja Z. Where there is no weighing scale: newborn nourishment and care in Pakistani Punjab. Womens Stud Int Forum. (2017) 60:128–35. 10.1016/j.wsif.2016.10.012

[39] Musoke D Boynton P Butler C Musoke MB. Health seeking behaviour and challenges in utilising health facilities in Wakiso district, Uganda. Afr Health Sci. (2014) 14:1046. 10.4314/ahs.v14i4.36 25834516 PMC4370086

[40] Fottrell E Tollman S Byass P Golooba-Mutebi F Kahn K. The epidemiology of “bewitchment” as a lay-reported cause of death in rural South Africa. J Epidemiol Community Health. (2012) 66:704–9. 10.1136/jech.2010.124305 21515546 PMC3402739

[41] Ashforth A Nattrass N. Ambiguities of “culture” and the antiretroviral rollout in South Africa. Soc Dyn. (2005) 31:285–303. 10.1080/02533950508628716

[42] Ahmed F Malik NI Shahzad M Ahmad M Shahid M Feng XL. Determinants of infant young child feeding among mothers of malnourished children in South Punjab, Pakistan: a qualitative study. Front Public Health. (2022) 10:834089. 10.3389/fpubh.2022.834089 35664102 PMC9160796

[43] Shahid M Cao Y Ahmed F Raza S Guo J Malik NI. Does mothers' awareness of health and nutrition matter? A case study of child malnutrition in marginalized rural community of Punjab, Pakistan. Front Public Health. (2022) 10:792164. 10.3389/fpubh.2022.792164 35211439 PMC8863129

