# Supplementary material for: Rural mothers' beliefs and practices about diagnosis, treatment, and management of children health problems: A qualitative study in marginalized Southern Pakistan
Source: Front Public Health. 2023 Jan 4;10:1001668. doi: 10.3389/fpubh.2022.1001668 (PMC9845559; doi:10.3389/fpubh.2022.1001668)
Supplement: Supplementary file 1 [file Data_Sheet_1.PDF]

Study: Rural Mothers‘ Diagnosis and Treatment Methods among Rural Mothers in a Marginalized District of South-Punjab, Pakistan

Under Research Project: Sociocultural Construction of Child and Mother Malnutrition in Rajanpur
(No. QAU-ASRB-2016-307; Date of approval 20-10-2016).

Semistructured Interview Guide

Sociodemographic Characteristics of Participants/Informant

Age___________________ Gender___________________

Occupation________________ Literacy level___________________

Household Income__________ Area/District___________________

Date___________________

Time on interview began__________ Time when interview ended__________

Name of the interviewer___________ Name of facilitator___________

Introduction

Today we want to discuss some issues related to different ways mothers and households observe diseases symptoms (diagnose), and respond to them (treatment).

<u>Questionnaire</u>

What are common types of childhood diseases and malnutrition? [Probe]

Does your household have access to the local health team (vaccinator, LHW, etc.)? [Probe]

Does the mother have access to the health professional? [Probe]

What is the people's capability to the quality and type of health facility? [Probe]

How is the quality of care and medicines are always available in remote public hospitals? Do low-income poor are stigmatized by the health staff? [Probe]

How is illness perceived or diagnosed? [Probe]

How is malnutrition assessed at the local level? [Probe] (anthropometric measurement, MUAC, weight, height, or other traditional methods used for judging if child is malnourished)

Do you have easy access to health and nutrition programs or have to face difficulties? [Probe]

Where do you get treatment for your children? Domestically, Herbal, medical, spiritual other [Probe] (quakes, bone settlers, etc.)

Where do you get treatment for a daughter, and where do you get treatment for a son? [Probe]

What types of infection did the child face, and how was treated? [Probe]

How are diarrhea and other infections diagnosed and treated by mothers? [Probe]

Why do you go there? [Probe] Do you mostly visit Spiritual healers (pir), or a medical doctor?

What Spiritual and Magico-Religious Healing Methods are commonly used by rural mothers? [Probe]

What are the benefits of vaccination, health education, prevention, and how this benefits children and pregnant and lactating mothers?

What are the etiologies of a disease? [Probe] What are locals' prototypes of explaining and analyzing illnesses? Does malnutrition is perceived as a contagious disease?

What sentences are recited by spiritual healing? [Probe] Quranic, Magic, other

What may be the main reasons behind informal methods of treatment? [Probe]

How far do inequalities (area, income,) construct health capacity, capability, and accessibility to health?

How does the literacy of mothers influence health status? How does household poverty influence health status?

How does community deprivation is linked to low access to formal healthcare?

How does mothers low social capital impact access to healthcare?

Final Note: Thank you very much for your participation, and cooperation, and answers to questions. Please add anything that was not asked about?
